# Assessment of sociodemographic and psychological well-being of primary school teachers

**DOI:** 10.47626/2237-6089-2022-0479

**Published:** 2024-02-06

**Authors:** Chinedu Ifedi Okeke, Moses Onyemaechi Ede, Fidelis Eze Amaeze

**Affiliations:** 1 Childhood Education University of the Free State Bloemfontein South Africa Childhood Education , University of the Free State , Bloemfontein , South Africa .; 2 Department of Educational Foundations Faculty of Education University of Nigeria Nsukka Enugu Nigeria Department of Educational Foundations , Faculty of Education , University of Nigeria , Nsukka , Enugu , Nigeria .

**Keywords:** Sociodemographic, gender, working status, psychological well-being, primary school, teachers

## Abstract

**Background:**

Teaching is very stressful and demanding and it intensifies psychological and related disorders compared to other occupations. Most teachers in Nigeria are battling emotional distress, burnout, depression, and anxiety due to excess workload, which has affected their psychological well-being. This study assessed the sociodemographic and psychological well-being of primary school teachers in Enugu State, Nigeria.

**Methods:**

This is a study with a cross-sectional design. The psychological well-being of 254 primary school teachers was assessed using a psychological well-being scale and a sociodemographic information inventory was also used to ascertain their personal information. The data collected were analyzed using chi-square, mean, standard deviation, and bivariate correlation statistics.

**Results:**

The results showed that the majority of primary school teachers experience unhealthy psychological well-being irrespective of age, gender, working status, qualification, and years of teaching experience. These teachers’ sociodemographic variables are significantly correlated with psychological well-being. Only location is not significantly correlated with psychological well-being.

**Conclusion:**

Therefore, this study concluded that primary school teachers’ psychological well-being is not positive or healthy and is significantly related to their sociodemographic characteristics.

## Introduction

The workload in teaching is very stressful and is compounded by many other issues that impact psychological wellbeing compared to other occupational areas. ^1^ These include emotional distress, burnout, depression, and anxiety. ^2 , 3^ This situation is particularly severe in the Nigerian context, as teachers, especially in primary schools, are disregarded, bonded into frustration and attrition. ^4^ A recent study conducted in Nigeria on teachers’ psychological well-being found that a high number of teaching staff in Nigeria are experiencing serious psychological distress. ^5^ Another study reported a 32.9% prevalence of psychological problems in teachers in Enugu state, Nigeria. ^6^ Instead of being regarded as critical stakeholders, pillars of development, and valuable assets for national development, ^7 , 8^ teachers in Nigeria are lured into facing ill health and psychological exhaustion. ^9 , 10^ This is to say that if the emotional state of teachers can be stabilized, there may be quality outputs. ^1^ Hence, it is disturbing that a good number of studies show that teachers’ experiences of fatigue leads to low productivity in teaching ^9 , 10^ and poor standards in the Nigerian educational system. ^1^ Some demographic variables (gender, educational qualification, work experience, and work status) have been identified as possible predictors of teachers’ poor psychological well-being. ^11 , 12^ However, it is worth noting that there is no existing theoretical support regarding this. ^1^

Theoretically, Carol Ryff’s psychological well-being model provides a powerful framework for analyzing and organizing one’s life, as well as for generating ideas for how to live better. According to Ryff, people should have a positive attitude about themselves, acknowledging and accepting many elements of themselves, including both good and terrible attributes, and feel positive about their prior lives. Having such feelings, people could have better psychological well-being. These characteristics show how happy an individual could be in the workplace.

Given these reasons, much attention has been paid to the psychology of teachers due to its importance. ^13^ This seems to have led to deeper development of psychological well-being. Psychological well-being is not only optimal psychological functioning but also the presence of psychological disturbances or ill health. ^1^

Past studies highlighted the need for more studies in the field of psychological well-being because there is limited literature on the well-being of schoolteachers. ^14^ The existing literature only focused on the psychological well-being of teachers at other levels of education or rather regular schoolteachers ^15 , 16^ and there seems to be a dearth of studies on the psychological well-being of primary school teachers in Nigeria. ^14^ Given the above worrisome state of psychological well-being of teachers in developing regions, we assessed the sociodemographic and psychological well-being of primary school teachers in Enugu State, Nigeria. To achieve the aforementioned aim, we hypothesized that a significant correlation exists between the sociodemographic variables and the psychological well-being of primary school teachers.

## Methods

### Design

This is a cross-sectional study.

### Study location

This study was conducted in Enugu state, Nigeria, specifically in the Enugu North Senatorial zone. In this area of study, there are both public and private primary schools. Public primary schools were investigated and we did not include private-owned schools as they may not have a specific system of operation.

### Information about study approval

Approval to conduct this study was obtained from the Faculty of Education, University of Nigeria, Enugu State Universal Basic Education, and the schools’ headmasters. This was followed by the completion of informed consent by the primary school teachers who were recruited after satisfying the inclusion criteria as specified in this study. The inclusion criteria include: 1) being a primary school teacher; 2) having been trained in teacher education institutes; 3) teaching within the study setting; and 4) being a confirmed staff member.

Some teachers were excluded based on the following reasons: 1) contract teachers who were parent-teacher association teachers; 2) teachers who were receiving medical attention were not present.

It was made clear to participants that they could withdraw from the study if they wished.

### Participants

We enrolled primary school teachers as participants. A total of 254 teachers who were officially recruited by the Government of Enugu State of Nigeria to teach in primary schools responded to the measurement tools used in the study. A convenience sampling method was adopted for selection of participants and study locations. This method was used for easy accessibility and proximity of the location. The participants were teachers from regular and special schools with primary sections. Of a total of 300 teachers, 254 teachers participated in the study, i.e., a 84.67% response rate. The participants were from urban 121 (47.6%) and rural 133 (52.4%) settings in the Nsukka Senatorial zone of Enugu State. Participants’ ages were as follows: 25 (9.8%) participants were younger than 25 years old, 87 (34.3%) were 26-35 years old, 84 (33.1%) were 36-54 years old, and 58 (22.8%) were 55 or older. Most of the participants were female, 177 (69.7%), and 77 (30.3%) were male. Some of the participants, 66 (26%), were single, most of them married, 171 (67.3%), a few of them were separated, 16 (6.3), and there was only one divorced participant, 1 (0.4%). For work status, 33 (13%) participants were lower cadre, 167 (65.4%) participants were middle cadre, and 54 (21.3) participants were upper cadre. While 107 (42.1%) participants had less than 10 years of experience, 105 (41.3%) had 10 to 20 years of experience, 35 (14.2%) had 21 to 30 years of experience, and six (2.4%) had 31 to 40 years of experience. In terms of educational qualifications, 115 (45.3%) participants had obtained their bachelor degrees, 54 (21.3%) participants had master’s degrees, 61 (24%) participants had a PhD, and 24 (9.4%) participants had other educational qualifications.

### Study measures

#### Psychological well-being

The present study used 24 items from the shortened version of Ryff’s ^15 , 16^ psychological well-being scale. These items sought to measure the six dimensions of personal growth, autonomy, environmental mastery, self-acceptance, purpose in life, and personal relations. To test the proposed six-factor structure, a principal component analysis with varimax rotation was computed. Three factors were extracted each with an eigenvalue greater than 1, and these factors related to positive self-evaluation, self-contentment, and sense of competence. This structure differed from the predicted six-dimensional structure reported in Ryff’s ^15 , 16^ psychological well-being scale. Given that the aim of this study was not to test the validity of the six dimensions but to obtain an overall measure of a teacher’s sense of psychological well-being, it was decided to compute an overall psychological well-being score. The combined psychological well-being scale had 28 items, after excluding two items referring to the dimensions of autonomy (“it is difficult for me to voice my opinions on controversial matters”) and self-acceptance (“my attitude about myself is probably not as positive as most people feel about themselves”) and revealed satisfactory internal consistency with α = 0.76.

The sociodemographic information inventory (SII) is a checklist used to ascertain the participants’ sociodemographic data with regards to gender, location, marital status, years of experience, and educational qualifications. This was constructed by the researchers of the present study. These sociodemographic components constitute the scope of participants’ personal data to which the present study was limited.

## Research procedures

The data were personally collected by the researchers. Data were collected at the various primary schools in the study setting. During data collection, participants were issued copies of instruments for completion. The questionnaires took 90 minutes to complete, 45 minutes per questionnaire. We ensured that data were collected shortly after working hours and data collection lasted 2 months.

## Data analysis

Shortly after collection, data were input to IBM SPSS Statistics. They were analyzed using frequency distributions and percentages to understand the sociodemographic characteristics of the participants. A bivariate correlation analysis was employed to test the correlations of relationships between variables. In this study, we adopted a real limit of number, stating that 21% and above was taken to be unhealthy or low psychological well-being while 20% and below was taken to be healthy psychological well-being.

## Results

Table 1 shows that 25 (9.8%) primary school teachers, below 25 years of age, experience healthy psychological well-being. It also shows that 87 (34.3%) teachers within the age range of 26-35, 84 (33.1%) within the age range of 36-54, and 58 (22.8%) within the age range of 46 and above experience unhealthy psychological well-being. Based on age, the results indicate that teachers within the age range of 26-35 years (n=87, 34.3%) experience unhealthy (low) psychological well-being more than other age groups. In terms of working status, 33 (13%) participants in a lower cadre experience healthy psychological well-being, however, 167 (65.7%) participants in a middle cadre, and 54 (21.3%) participants in a higher cadre experienced unhealthy (low) psychological well-being. This is an indication that teachers in the middle cadre experience low psychological well-being more than other counterparts (n=167, 65.7%) Regarding educational qualifications, 115 (45.3%) participants with bachelor’s degrees, 54 (21.3%) with master’s degree, and 61 (24%) with a PhD experience unhealthy psychological well-being. On the other hand, only 24 (9.4%) participants with other different qualifications experienced psychological well-being. This demonstrates that teachers with bachelor’s degree experience unhealthy psychological well-being more compared to others. For the teaching experience, the results show that 107 (42.1%) teachers with less than 10 years’ experience and 105 (41.3%) teachers with 10 to 20 years’ experience had unhealthy psychological well-being, while 6 (14.2%) teachers with 21 to 30 years’ teaching experience and six (2.4%) teachers with 31 to 40 years’ teaching experience had healthy psychological well-being. The majority of primary school teachers who had less than 10 years’ teaching experience were rated as experiencing unhealthier psychological well-being. Table 1 also shows that 177 (69.7%) females and 77 (30.3%) male teachers experience unhealthy psychological well-being. However, female teachers (n=177, 69.7%) experience more unhealthy psychological well-being compared to male teachers (n=77, 30.3%). Regarding location, Table 1 shows that 133 (52.4%) teachers in rural locations and 121 (47.6%) urban teachers experience unhealthy psychological well-being The results also indicated a higher rate of unhealthy psychological well-being among teachers in rural locations (n=133, 52.4%) than those in urban locations (n=121, 47.6%).

**Table 1:** Descriptive analysis of the level of psychological well-being according to sociodemographic variables

	n (%)	PWS total score (mean ± SD)
Age, years		
Under 25	25 (9.8)	100.01 ± 6.77
26-35	87 (34.3)	104.88 ± 27.58
36-54	84 (33.1)	107.38 ± 18.18
46 and over	58 (22.8)	109.46 ± 15.03
Work status		
Lower cadre	33 (13.0)	107.09 ± 25.43
Middle cadre	167 (65.7)	105.65 ± 21.22
Upper cadre	54 (21.3)	107.68 ± 15.67
Qualification		
Bachelor’s degree	115 (45.3)	108.90 ± 17.34
Master’s degree	54 (21.3)	107.78 ± 21.34
PhD	61 (24.0)	97.07 ± 22.55
Others	24 (9.4)	113.65 ± 23.22
Marital status		
Single	66 (26.0)	108.08 ± 25.06
Married	171 (67.3)	105.76 ± 19.88
Separated	16 (6.3)	104.24 ± 4.76
Divorced	1 (0.4)	107.02 ± 00.00
Years of experience		
Less than 10	107 (42.1)	106.60 ± 24.24
10 to 20	105 (41.3)	105.13 ± 18.02
21-30	36 (14.2)	109.92 ± 17.67
31 to 40	6 (2.4)	98.38 ± 12.00
Gender		
Male	77 (30.3)	103.81 ± 18.31
Female	177 (69.7)	107.34 ± 21.65
Location		
Urban	121 (47.6)	101.72 ± 18.42
Rural	133 (52.4)	110.41 ± 21.88

PWS = personal well-being score.

The results in Table 2 show that the primary school teachers’ psychological well-being has significant positive associations with age (r = 0.127*, p ˃ 0.044), working status (r = 0.016, p ˃ 0.798), years of experience (r = 0.000, p ˃ 0.995), and gender (r = 0.079, p ˃ 0.212). Only location is not significantly correlated with psychological well-being, r = 0.210**, p ˃ 0.001. On the other hand, Table 2 shows that the educational qualification and marital status of the primary school teachers have significant negative correlations with psychological well-being (r = -0.094, p ˃ 0.136; r = -0.052, p ˃ 0.410). This indicates that increases in age, years of experience (teaching experience), and gender are associated with increased in the psychological well-being of the primary school teachers.

**Table 2:** Bivariate analysis of relationships among psychological well-being and sociodemographic variables

	Age	Working status	Qualification	Marital status	Years of experience	Gender	Location	PWS
Age								
Pearson correlation	1	0.296*	-0.339*	0.162*	0.542*	0.065	-0.039	0.127 ^†^
Sig. (2-tailed)		0.000	0.000	0.010	0.000	0.303	0.534	0.044
N		254	254	254	254	254	254	254
Working status								
Pearson correlation		1	-0.082	0.210*	0.436*	0.124 ^†^	0.027	0.016
Sig. (2-tailed)			0.192	0.001	0.000	0.049	0.665	0.798
n			254	254	254	254	254	254
Qualification								
Pearson correlation			1	-0.091	-0.193*	0.018	0.024	-0.094
Sig. (2-tailed)				0.149	0.002	0.775	0.704	0.136
n				254	254	254	254	254
Marital status								
Pearson correlation				1	0.210*	-0.211*	-0.370*	-0.052
Sig. (2-tailed)					0.001	0.001	0.000	0.410
n					254	254	254	254
Years of experience								
Pearson correlation					1	0.145 ^†^	-0.062	0.000
Sig. (2-tailed)						0.021	0.325	0.995
n						254	254	254
Gender								
Pearson correlation						1	0.143 ^†^	0.079
Sig. (2-tailed)							0.023	0.212
n							254	254
Location								
Pearson correlation							1	0.210*
Sig. (2-tailed)								0.001
n								254
PWS								
Pearson correlation								1
Sig. (2-tailed)								
n								

PWS = personal well-being score.

* Correlation is significant at the 0.01 level (two-tailed).

^†^ Correlation is significant at the 0.05 level (two-tailed).

## Discussion

The purpose of this study was to assess the psychological well-being of primary school teachers in Enugu State, Nigeria. The results showed that the majority of primary school teachers experience unhealthy psychological well-being irrespective of age, gender, working status, or years of teaching experience. The results also showed that age has a significant positive association with the psychological well-being of primary school teachers. Only location is not significantly correlated with psychological well-being. The results further showed that the educational qualifications and marital status of the primary school teachers have significant negative correlations with psychological well-being.

The results indicated that the majority of primary school teachers experience unhealthy psychological well-being regardless of their age, gender, educational qualifications, working status, or years of teaching experience. This findings agrees with a past study that demonstrated that teachers’ well-being is known to be subject to frequent negative public health issues (stress and burnout) facing teachers. ^17^ Contrary to our expectation, a past study also found that psychological well-being has no significant relationship with work experience. ^18^ This does not confirm that teachers in most primary schools feel distressed in the course of delivering their teaching responsibilities, thereby affecting their work balance. Like the present study, Gray et al., ^19^ stated that teachers do not enjoy occupational wellness and satisfaction as they are faced with serious work-related stress. Given that their low psychological well-being is not positive for teachers’ health, previous studies constantly reported that they are highly vulnerable to amplified psychological disturbances. ^20 , 21^ Teachers could not achieve positive psychological well-being as the conditions in the workplace do not permit them to establish a warm psychological relationship, ^22^ thereby disrupting their occupational growth. For example, if the school climate is harsh, perhaps it leads to organizational misbehaviors among teachers. ^23^ Experiencing positive psychological well-being increases commitment and turnover among teachers. ^24^

As admitted by the primary school teachers in this study, a Spanish study showed that teaching is a demanding task and teachers were delivering the tasks in difficult working conditions. ^25^ In line with our findings, past studies showed that teachers with negative psychological mental health may find it more difficult to deliver duties entrusted to them ^26 , 27^ and that negative psychological mental health increases poor teacher-student relationships. ^28^

Considering the results of the present study as related to the teachers’ sociodemographic factors, past empirical evidence demonstrated that age, gender, years of teaching experience, and working status moderate the psychological well-being of teachers. ^18^ Similarly, Hascher and Waber ^17^ reported that teachers’ competence negatively predicts the well-being of younger and older teachers.

In terms of gender, a past study showed that gender does not significantly relate to psychological well-being, ^29^ showing that the psychological well-being of the teachers is the same regardless of their gender. Similarly, the outcomes of the current study support Amran and Khairiah, ^30^ who showed a significant relationship between gender and general well-being. The significant correlation extends to other symptoms of psychological well-being such as depression and anxiety. ^31 , 32^

Confirming the findings of the present study, Matud et al. ^33^ highlighted that there is a need for critical examination of gender-related studies that showed a significant difference between psychological well-being and gender.

## Conclusion

This study concluded that the majority of primary school teachers experience unhealthy psychological well-being irrespective of age, gender, working status, qualification, or years of teaching experience. These teachers’ sociodemographic variables are significantly correlated with psychological well-being. Only location is not significantly correlated with psychological well-being.

### Practice implications

The outcomes of the present study have far-reaching practice implications for experts in school counseling, occupational health, and education. As the results indicated, practicing school counselors should be aware that teachers at the primary level appear to be dissatisfied, leading to poor psychological functioning. These unhealthy conditions have been demonstrated in their perception of the teaching job. Given this, counselors should seek effective occupational health therapy to cushion the adverse effects of their negative psychological functioning that have affected their well-being.

Experts in the field of occupational health should also advocate for a paradigm shift in policies related to teachers’ satisfaction. In doing that, there should be a balance between the hedonistic and eudemonic aspects of teachers’ psychological well-being. It is possible, therefore, that if teachers’ well-being is taken care of, there would be increased turnover, presentism, productivity, and commitment.

Teachers’ psychological experience counts in terms of interpersonal and intrapersonal relationships in the school. Therefore, psychologists in the field of education should look in depth to identify psychoeducational measures and interventions that could be used to assist teachers with negative psychological well-being. If a teacher is not happy within himself, it could affect other things he does in the workplace.

### Limitations

There are some methodological limitations noted in the course of carrying out this study. The specific dimensions of psychological well-being were not measured. If this had been done, it would have been possible to explore the specific dimensions in which teachers are dissatisfied. Moreover, we could not demonstrate how teachers’ psychological well-being correlates with the pupils’ academic performance or how this could affect the mental well-being of the pupils. Therefore, we implore future studies not to concede to these limitations.

## References

[1] Mabekoje AS (2003). Psychological well-being among Nigerian teachers: a discriminant function analysis. J Res Couns Psychol.

[2] Ede MO, Adene FM, Okeke CI, Mezieobi DI, Isiwu EN, Abdullahi Y (2021). The effect of rational emotive behaviour therapy on post-traumatic depression in flood victims. J Ration Emot Cogn Behav Ther.

[3] Ede MO, Okeke CI, Adene FM, Areji AC (2023). Perceptions of work value and ethical practices amongst primary school teachers, demographics, intervention, and impact. Psychol Rep.

[4] Ofili AN, Usiholo EA, Oronsaye MO (2009). Psychological morbidity, job satisfaction and intentions to quit among teachers in private secondary schools in Edo-State, Nigeria. Ann Afr Med.

[5] Ozoemena EL, Agbaje OS, Ogundu L, Ononuju AH, Umoke PCI, Iweama CN (2021). Psychological distress, burnout, and coping strategies among Nigerian primary school teachers: a school-based cross-sectional study. BMC Public Health.

[6] Okwaraji FE, Aguwa EN (2015). Burnout, psychological distress, and job satisfaction among secondary school teachers in Enugu, South-East Nigeria. J Psychiatry.

[7] Khani R, Mirzaee A (2015). How do self-efficacy, contextual variables and stressors affect teacher burnout in an EFL context?. Educ Psychol.

[8] Pishghadam R, Derakhshan A, Zhaleh K, Al-Obaydi LH (2023). Students’ willingness to attend EFL classes with respect to teachers’ credibility, stroke, and success: a cross-cultural study of Iranian and Iraqi students’ perceptions. Curr Psychol.

[9] Benevene P, De Stasio S, Fiorilli C (2020). Editorial: well-being of schoolteachers in their work environment. Front Psychol.

[10] Jin Y, Zhang LJ, MacIntyre PD (2020). Contracting students for the reduction of foreign language classroom anxiety: an approach nurturing positive mindsets and behaviors. Front Psychol.

[11] Alarcon GM, Eschleman KJ, Bowling NA (2009). Relationships between personality variables and burnout: a meta-analysis. Work Stress.

[12] García-Arroyo JA, Osca Segovia A, Peiró JM (2019). Meta-analytical review of teacher burnout across 36 societies: the role of national learning assessments and gender egalitarianism. Psychol Health.

[13] Li S (2021). Psychological wellbeing, mindfulness, and immunity of teachers in second or foreign language education: a theoretical review. Front Psychol.

[14] Olagunju AT, Akinola MA., Fadipe B, Jagun OO, Olagunju TO, Akinola OO (2021). Psychosocial wellbeing of Nigerian teachers in special education schools. J Autism Dev Disord.

[15] Ryff CD (1989). Beyond Ponce de Leon and life satisfaction: new directions in quest of successful aging. Int J Behav Dev.

[16] Ryff CD (1989). In the eye of the beholder: views of psychological well-being among middle-aged and older adults. Psychol Aging.

[17] Hascher T, Waber J (2021). Teacher well-being: a systematic review of the research literature from the year 2000–2019. Educ Res Rev.

[18] Terry DJ, Nielsen M, Perchard L (1993). Effects of work stress on psychological well-being and job satisfaction: the stress-buffering role of social support. Aust J Psychol.

[19] Gray C, Wilcox G, Nordstokke D (2017). Teacher mental health, school climate, inclusive education and student learning: a review. Can Psychol.

[20] Stansfeld SA, Rasul F, Head J, Singleton N (2011). Occupation and mental health in a national UK survey. Soc Psychiatry Psychiatr Epidemiol.

[21] Kidger J, Brockman R, Tilling K, Campbell R, Ford T, Araya R (2016). Teachers’ wellbeing and depressive symptoms, and associated risk factors: a large cross-sectional study in English secondary schools. J Affect Disord.

[22] Harding S, Morris R, Gunnella D, Ford T, Hollingworth W, Tilling K (2019). Is teachers’ mental health and wellbeing associated with students’ mental health and wellbeing?. J Affect Disord.

[23] Grayson JL, Alvarez HK (2008). School climate factors relating to teacher burnout: a mediator model. Teach Teach Educ.

[24] Office for Standards in Education (Ofsted) (2019). The education inspection framework.

[25] United Nations Educational Scientific and Cultural Organization (UNESCO) TVET Systems and Labour Markets. UNESCO COVID-19 education response: education sector issue notes.

[26] Kidger J, Gunnell D, Biddle L, Campbell R, Donovan J (2010). Part and parcel of teaching? Secondary school staff’s views on supporting student emotional health and well-being. Br Educ Res J.

[27] Jennings PA, Greenberg MT (2009). The prosocial classroom: teacher social and emotional competence in relation to student and classroom outcomes. Rev Educ Res.

[28] Sisask M, Värnika P, Värnik A, Apter A, Balazs J, Balint M (2013). Teacher satisfaction with school and psychological well-being affects their readiness to help children with mental health problems. Health Educ J.

[29] Kamaruzaman M, Surat S (2021). Teachers’ psychological well-being during Covid-19 pandemic. Int J Acad Res Bus Soc Sci.

[30] Amran H, Khairiah K (2014). Hubungan tekanan terhadap kesejahteraan umum dalam kalangan penjawat awam di pejabat setiausaha kerajaan negeri Pahang. Humanitas (Universitas Ahmad Dahlan Fak Psikol).

[31] Casey L, Rebecca M (2011). Stress and wellbeing in Australia in 2011: a state-of-the-nation survey. 2011 National Psychology Week research survey. InPsych.

[32] Johari H, Pusphavalli R (2020). Hubungan di antara konsep kendiri dan kesejahteraan hidup di Kalangan Remaja Akhir.

[33] Pilar Matud M, López-Curbelo M, Fortes D (2019). Gender and psychological well-being. Int J Environ Res Public Health.

